# The Role of Frailty in Predicting 3 and 6 Months Functional Decline in Hospitalized Older Adults: Findings from a Secondary Analysis

**DOI:** 10.3390/ijerph18137126

**Published:** 2021-07-03

**Authors:** João Tavares, Pedro Sa-Couto, João Duarte Reis, Marie Boltz, Elizabeth Capezuti

**Affiliations:** 1 School of Health Sciences, University of Aveiro, 3810-193 Aveiro, Portugal; 2 Center for Health Technology and Services Research, 3810-193 Aveiro, Portugal; 3 Health Sciences Research Unit: Nursing (UICISA: E), 3000-232 Coimbra, Portugal; 4 Department of Mathematics (DMAT), University of Aveiro, 3810-193 Aveiro, Portugal; p.sa.couto@ua.pt (P.S.-C.); jduarte.reis@ua.pt (J.D.R.); 5 College of Nursing, Pennsylvania State University, University Park, PA 10927, USA; mpb40@psu.edu; 6 School of Nursing, Hunter College of the City University of New York, New York, NY 10010, USA; ec773@hunter.cuny.edu

**Keywords:** aged, frail older adults, frailty, hospitalization, functional status, adverse effects

## Abstract

Frailty represents one of the most relevant geriatric syndromes in the 21st century and is a predictor of adverse outcomes in hospitalized older adult, such as, functional decline (FD). This study aimed to examine if frailty, evaluated with the Frailty Index (FI), can predict FD during and after hospitalization (3 and 6 months). Secondary data analysis of a prospective cohort study of 101 hospitalized older adults was performed. The primary outcome was FD at discharge, 3 and 6 months. The FI was created from an original database using 40 health deficits. Functional decline models for each time-point were examined using a binary logistic regression. The prevalence of frailty was 57.4% with an average score of 0.25 (±0.11). Frail patients had significant and higher values for functional decline and social support for all time periods and more hospital readmission in the 3 month period. Multivariable regression analysis showed that FI was a predictor of functional decline at discharge (OR = 1.07, 95% CI = 1.02–1.14) and 3-month (OR = 1.05, 95% CI = 1.01–1.09) but not 6-month (OR = 1.03, 95% CI = 0.99–1.09) follow-up. Findings suggest that frailty at admission of hospitalized older adults can predict functional decline at discharge and 3 months post-discharge.

## 1. Introduction

Frailty represents one of the most relevant geriatric syndromes. Various definitions and approaches are represented in the literature [1]. An international consensus group of experts defined frailty as “a clinical state in which there is an increase in an individual’s vulnerability for developing an increased dependency and/or mortality when exposed to a stressor” [2] (p. 329). Two noteworthy perspectives on frailty emerged in 2001. One described frailty as a clinical syndrome with physical characteristics [3] that can be considered a pre-disability [4]. The other described frailty as a state of accelerated deficit accumulations [5]. Both perspectives agree that frailty is a multifactorial condition, that compromises physiological reserve and is a major contributor to morbidity and mortality among older adults, especially in the cohort group ≥70 years. Reviews clearly demonstrated that frailty was a predictor of adverse outcomes including disability, falls, delirium, hospitalization and mortality [5,6,7]. The majority of these studies were conducted with community-dwelling older adults or long-term care residents, rather than hospitalized older adults. However, the prevalence of frailty in acute care setting was significantly higher, with a mean prevalence of 49% and a range from 34% to 69%, varying by type of ward (*n* = 112 articles) [7]. Additionally, this variability was influenced by the type of instrument used in evaluation and the characteristics of the population.

The higher level of frailty among hospitalized older adults is explained by the vulnerability to health crises that result in greater use of health resources, specially, the emergency department (ED) and hospitals. Hospitalization represents a stress event for older adults, often resulting in cognitive decline, fall, delirium, infections, pressure injuries and mortality [8]. Among hospitalized older adults, frail older persons have an increased risk of adverse outcomes compared to the robust patients [6,9,10]. The most frequent outcomes predicted by frailty are: mortality (84%), longer in-hospital length of stay (LOS) (73%), institutionalization (93%), and in-hospital complications, such as, delirium, falls and functional decline (69%) [7]. Recent studies emphasized the association of frailty with hospital readmission within 30 days [9,11] and in the three year follow-period [10]. Additionally, frailty is linked to the increased likelihood of ED readmission (odds ratio (95% CI), 1.24 [1.11–1.37]) [12].

Despite the high prevalence of functional decline in hospitalized older adults (30–60%) [13] and its association with increased mortality [14,15] and lower quality of life, this outcome is less commonly studied in research. In a scoping review that examined frailty in the acute care setting, Theou et al. [7] found that functional decline and quality of life only account for 4% of all outcomes reported in hospital-based studies. Similar results were reported in a systematic review and meta-analysis of frailty as a predictor of adverse outcomes in the hospitalization of older adults. The results showed that frail and pre-frail older adults have 1.32 (95% CI: 1.04–1.67) and 1.51 (95% CI: 1.05–2.17), respectively, more risk of functional decline during hospitalization as compared with non-frail older adults [6]. The authors emphasized that functional decline was poorly assessed as a primary outcome (5 out of 28 papers), due to the difficulty of determining this decline, since it was not evaluated at different times, including upon admission and discharge. The majority of studies defined functional decline as occurring between admission and discharge and used various tools to evaluate physical function, such as the Katz Index, Barthel Index or clinical judgments determined by trained nurse or geriatrician. One study, however, noted frailty among those with higher functional impairment after hospitalization [16]. Other studies of functional decline in hospitalized older adults have reported that a significant number of patients did not return to baseline functional status after hospitalization [17,18,19], but the link with frailty was not examined. To the best of our knowledge, no previous studies have examined frailty as a predictor of functional decline in hospitalized older adults in follow-up periods after discharge.

## 2. Materials and Methods

### 2.1. Aims

This study aimed to examine if frailty, measured with the Frailty Index (FI), can predict functional decline during and after hospitalization (3 and 6 months after discharge). We hypothesized that functional decline of hospitalized older adults can be predicted at discharge and 3 and 6 months follow-up period after discharge, based on their frailty condition along with other explanatory variables (see section on “variables”).

### 2.2. Design

A secondary analysis was performed of data from a prospective cohort study conducted between April and October 2016 that included a comprehensive geriatric assessment performed in the first 48 h of hospital admission. Functional decline was assessed at three timepoints (discharge, 3 months, and 6 months after discharge). The Ethics Commission of the hospital approved the study (No. 065–14). The participation was voluntary, and all participants signed an informed consent form.

### 2.3. Study Setting and Sample

The study was conducted in four internal medicine wards in a central public university hospital in the central region of Portugal. In the primary study the sample size was calculated with G-Power (*n* = 102). Inclusion criteria included 70 or more years old with the capacity to complete the questionnaire. Exclusion criteria were those transferred from the intensive care unit, terminal, or neurodegenerative disease, or score 0 in Katz Index (totally dependent, and hospitalized less than 48 h.

One hundred and seventeen participants enrolled in the initial phase, but 16 were lost to follow-up (either died or transferred to other units). The final sample included 101 hospitalized older adults.

### 2.4. Variables

#### 2.4.1. Primary Outcomes

Functional decline was the primary outcome in this study and assessed with the Katz Index (dichotomous scale, classified as dependent = 0 or independent = 1) at two weeks prior to admission (baseline), discharge (t0), 3 (t1) and 6 (t2) months post-discharge. Functional decline was defined as a loss of at least one point on the total Katz Index scores, calculated at each timepoint.

#### 2.4.2. Secondary Outcomes

Telephone follow-up was performed 3 and 6 months after discharge with dichotomous questions (yes or no) about hospital readmissions and ED visits (90 and 180 days) and use of social support (a single question asking if the older adult was receiving informal or formal support at home.)

#### 2.4.3. Explanatory Variables

In the primary study, a large number of variables were measured, such as sociodemographic (e.g., age, gender, marital status, education level) and clinical characteristics. The clinical variables included comorbidity (Charlson Comorbidity Index), medications, cognition, affective status, sensory ability (visual and auditory), fear of falling, pain, delirium, sleep, risk of falling, risk of pressure injuries and weight loss, and are described elsewhere [20]. These variables were used to create the exposure variable, FI. Following Searle and colleague’s standard procedure [21], we used 40 health deficits from the initial assessment (Table S1). Because the majority of deficits was dichotomous (0 or 1), ordinal variables were also dichotomized because of the negligible impact on the performance of the FI [22]. The FI was calculated by summing the deficits present and dividing by the total number of possible deficits. For example, a patient who demonstrated 10 deficits out of 40 possible, scored a FI of 0.25. We represented frailty, measured by the FI in two ways (continuous and categorical), using the approach reported in other studies [10,23]. The FI is a continuous score ranging from 0 to 1, labeled as FIcont and as a categorical variable (with a binary classification of 0 or 1 for non-frail and frail conditions, respectively), and labeled as FIcat using the cut-off of ≥0.25 to indicate frailty [24]. Only the variables not encoded directly in the FI were considered as predictors of functional decline during and after hospitalization (Table S1).

### 2.5. Procedure

In the primary study, most measurements were obtained by three registered nurses by in-person interview (upon hospital admission, between the 4th and 5th day of hospitalization, and at discharge) and by telephone 3 and 6 months following hospital discharge. Other Data were extracted from the electronic medical record. From the original database, the researcher (JT) conducted an analysis of the possible health deficits to ensure that prevalence of all deficits included in FI is no lower than 1% [24]. FI was calculated with the recoded variables.

### 2.6. Analysis

Patients’ admission characteristics and follow-up results were characterized by FI status. For FIcat, frequency and percentage were used and the Chi-squared test (including Yates’s correction for continuity for 2 × 2 contingency tables) for testing association between variables was applied. For the continuous presentation of FI (FIcont), means (M) with standard deviations (SD) were used. A parametric one-way ANOVA was applied to compare difference group means. Despite the verification of homogeneity tests (Levene test), the residuals’ normality was not verified for most cases (by visual inspection of Q–Q plots). Then, a non-parametric ANOVA (Kruskal-Wallis test) was applied. The statistical results achieved for the non-parametric test were identical to the ones presented by the parametric ANOVA. The correlation between two continuous variables was tested using the Spearman test.

Functional decline for each time-point: t0, t1 and t2 was studied using a binary logistic regression. Significant variables on univariable analysis were included in the multivariable model. The results were shown through the odds ratios (OR) and their 95% confidence interval (95% CI), and the area under curve (AUC) is presented for the multivariable models. Finally, the Likelihood ratio test and the Hosmer-Lemeshow test for goodness-of-fit were verified and no evidence of multicollinearity and overdispersion were found in any of the purposed models.

The statistical analysis was performed using R (v3.6.1) in RStudio (v1.3.1093) using the “foreign”, “car”, “pROC”, “lmtest”, “ResourceSelection” and “DescTools” packages. A *p*-value ˂ 0.05 was considered significant.

## 3. Results

The sample included 101 hospitalized older adults, 53.3% female, with a mean age of 82.47 ± 6.57 years. According to the variable “FIcat”, 58 patients (57.4%) were identified as frail. The FIcont had an average score of 0.25(±0.11), median of 0.25 and minimum and maximum of 0.05 and 0.5, respectively. The histogram of the FIcont variable showed a symmetrical and platykurtic distribution (skewness (M ± SE): 0.00 ± 0.2; kurtosis (M ± SE): −1.03 ± 0.54).

The sample characteristics by their frailty status is shown in Table 1. The analysis of these characteristics, with frailty as a categorial variable, demonstrated a significant relationship with older age (≥80 years), those with a lower education level, multimorbidity, previous hospitalization and at risk for functional decline (Identification of Seniors at Risk-Hospitalized Patients-ISAR-HP cutoff > 2) compared to non-frail older adults.

The analysis of frailty as a continuous variable indicated a significant relationship with those who were female, older, less educated, not married, institutionalized, previously hospitalized and with multimorbidity. The correlation presented moderate to strong dependencies between FIcont and age (r = 0.39), number of drugs (r = 0.44) and ISAR-HP (r = 0.68).

### 3.1. Outcomes

Functional decline at 3 months post hospital discharge occurred in 33 patients. Twenty older adults were rehospitalized, and 38 had ED visits. Among older adults discharged to home (*n* = 83), 79.7% needed social support. Compared with non-frail older adults, functional decline among frail older adults between baseline and discharge was significantly higher (69.1% vs. 30.9%). Patients with functional decline had higher FI means as compared to those that did not decline (0.29 ± 0.1 vs. 0.21 ± 0.11, *p* < 0.001). Similar results were found in those with functional decline at 3 months compared to baseline (*p* = 0.002) and discharge (*p* = 0.005) (Table 2). A higher frailty score was associated with a higher rate of hospital readmission (0.24 ± 0.11 vs. 0.31 ± 0.09, *p* = 0.008) and more need for social support (0.13 ± 0.06 vs. 0.24 ± 0.1, *p* < 0.001). There are no significant differences between frailty status and ED visits during 3 months following discharge.

Outcomes based on frailty status 6 months post discharge are reported in Table 3. During this period 21 patients had functional decline. Thirty-three were treated in the ED and 21 had a hospital readmission. Among older adults discharged to home (*n* = 83), 84% needed social support. Compared with non-frail older adults, functional decline was significantly higher (72.7% vs. 27.3%) in frail individuals. Patients that experienced functional decline had higher FI scores as compare to those that did not decline (0.28 ± 0.1 vs. 0.23 ± 0.12, *p* = 0.023). Similar results were found in functional decline between 6-month follow-up and discharge (0.23 ± 0.11 vs. 0.30 ± 0.09, *p* = 0.015) (Table 3). A higher score of frailty was associated with older people who needed more social support (0.12 ± 0.04 vs. 0.24 ± 0.11, *p* = 0.003). There were no significant differences between frailty status and hospital readmission or ED visits at the 6-month timepoint.

### 3.2. Multivariable Logistic Regression

#### 3.2.1. Functional Decline: Baseline to Discharge

The univariable analyses for FD between baseline–discharge showed that most of the variables considered were associated with functional decline (Table 4), with the exception of “level of education”, “living condition”, “multimorbidity”, “length of stay”, and “number of medicines”. The multivariable regression indicated that functional decline during the hospitalization was positively significantly associated with frailty (OR = 1.07, 95% CI = 1.02–1.14), not being married (OR = 5.12, 95% CI = 1.79–16.23), and previous hospitalization (OR = 4.98, 95% CI = 2.05–13.14). The AUC was 0.72 for this model, representing a 72% chance that the purposed model will be able to distinguish between positive functional decline and negative functional decline.

#### 3.2.2. Functional Decline: Baseline to 3 Months Post-Discharge

According to the univariable analyses, functional decline from baseline to 3 months post-discharge was significantly correlated to: being frail (dichotomous categorical: OR = 3.04, 95% CI = 1.25–7.95; continuous presentation: OR = 1.07, 95% CI = 1.02–1.12) and age (OR = 1.14, 95% CI = 1.06–1.24). The multivariable regression analysis showed that functional decline was positively associated with frailty (OR = 1.05, 95% CI = 1.01–1.09) and age (OR = 1.11, 95% CI = 1.04–1.20). The AUC was 0.68 for this model (Table 5).

#### 3.2.3. Functional Decline: Baseline to 6 Months Post-Discharge

The univariable analysis indicated that the following variables were significantly associated with functional decline: being frail (categorical presentation: OR = 3.09, 95% CI = 1.25–8.19; continuous presentation: OR = 1.05, 95% CI = 1.01–1.10), needing social support at admission (OR = 4.06, 95% CI = 1.29–13.78), and age (OR = 1.09, 95% CI = 1.01–1.17). The multivariable regression analysis of functional decline indicated that the risk significantly increased only with social support need (OR = 3.28, 95% CI = 1.25–9.09). Of notice, despite its non-significance, is the increased functional decline risk associated to the variables: frailty (OR = 1.03, 95% CI = 0.99–1.09) and age (OR = 1.07, 95% CI = 0.99–1.18). The AUC was 0.68 for this model (Table 6).

## 4. Discussion

Both hospitalization and frailty are associated with adverse outcomes in older adults. We found the prevalence of frailty (57.4%) and the mean frailty (continuous variable) (0.25 ± 0.11) in our Portuguese sample higher compared to previous studies in acute care settings conducted in western countries (48.5%) [7] and Asian countries, such as, China [10] and Vietnam [25] at 49.7% and 31.9%, respectively. Definition and assessment of frailty, methodological design and sample selection could influence the difference in prevalence rates. In our study, only older adults age 70 or more years were included. This criterion is relevant to understand our results because we found a strong correlation between advanced age and frailty [5]. The factors associated with frailty (older age, female, lower education, not married, multimorbidity and polypharmacy) were similar to those reported in several review articles and were associated with the onset or progression of frailty [26,27,28].

The results confirm, in part, our hypotheses that functional decline post-hospitalization can be predicted by frailty. In the univariable model, frailty was a predictor for functional decline at all timepoints alongside with other factors previously reported in other studies of functional decline, such as, being female, unmarried status, higher age, lower social support and previous hospitalization [29,30,31]. However, after adjustment for co-variates, frailty continued to be a significant predictor of risk for functional decline at discharge (OR = 1.07) and 3-month follow-up (OR = 1.05). Despite the non-significance found in the prediction of frailty for functional decline at 6 months (OR = 1.03), the greater risk was associated with more frail older adults. Our findings are in line with previous studies that highlight frailty as an independent risk factor for functional decline [6] and reinforce the importance of this syndrome as a predictor of adverse outcomes in hospitalized older adults. In the systematic review, among the 19 cohort studies, only five address functional decline between hospital admission and discharge [6]. Our findings, from a prospective cohort study with a follow-up three and six months after discharge, demonstrated the impact of frailty, not only during, but beyond hospitalization. Unlike most other studies [7], we examined functional decline from baseline function (two weeks prior to hospitalization) to discharge. Pre-admission functional decline can occur independently of co-morbidity or acute disease [32], and is an important predictor of post-acute functional status [33], with relevant impact for the patients, informal caregivers, families and society.

In Portugal, the percentage of FD at discharge, 3 month and 6 month follow-up were 54.5%, 35.5% and 37.9%, respectively [17]. Intervention to prevent functional decline in hospitalized older adults, must consider the frailty condition. These findings reinforce the need for screening or assessment of frailty in the older adult’s hospital admission and target interventions to manage frailty, in order to improve functional status and mitigate the potentially negative impact of hospitalization. In Portuguese hospitals screening and assessment of frailty is not a common practice, despite the recommendation of the importance of this by the task force of the International Conference of Frailty and Sarcopenia Research [4]. The high occurrence of functional decline and frailty among hospitalized older adults indicates the urgent need to implement best practice in the management of this syndrome.

Previous hospitalization (OR = 7.9) and need for social support (OR = 3.31) were risk factors not usually found in the literature and were significant factors for functional decline at discharge and 6 month post-discharge, respectively. One possible explanation could be the fact that previous hospitalization increased the functional decline and/or the progression of frailty, resulting in the need for more social support to provide care, and increased the risk of subsequent hospitalization. The results of meta-analysis identify frailty as a major factor predicting the risk for hospitalization in older persons [34]. In our results frail older patients have higher rate of hospital readmission at 3-month follow-up. This could represent a vicious cycle between the hospitalization-associated disability, social support and frailty. Possibly, frail older people are hospitalized more frequently, which in turn puts them at risk of functional decline due to the hospitalization itself and/or worsening of frailty status that precipitate the cascade of functional decline [4]. Interventions that interrupt this cycle are crucial to stall, slow or reverse these conditions. Further studies should examine the link between previous hospitalization, FD, frailty and social support, especially since these factors are potentially modifiable.

Frail older adults showed increased risk for 90-day hospital readmission and the need for more social support. These findings were supported in previous studies that report that frail patients have more risk for adverse outcomes compared to robust patients [6,9,10]. Results related to 30-day readmissions corroborate previous studies [9,11]; the majority of the studies examined only the 30 day hospital readmission. Some studies reported that readmission within 90 days of hospitalization for heart failure [35] and chronic obstructive pulmonary disease [36] can be predicted by frailty, as was found in our findings. Frail older adults have more hospital readmission at long (7–12 months) and very long term (over 2 years) [6], but more studies are need to obtain robust evidence.

The increased need for social support resulting from hospitalization and/or frailty could represent major burden for older adults, their family caregivers, and society in general. Our findings underscore the value of identifying frailty in order to identify older adults who need additional support services [1]. Typically, studies report the discharge destination, such as community, post-acute-care, long-term care institution. Among the older adults who are discharged to the community little information is reported about the need for social support. Our findings highlight the increased need for this support in more frail, community-based persons. Further investigation will be relevant to best characterize this support and the impact on quality of life and family/caregivers’ burden.

In this study frailty was not associated with ED visits. In other research, it has been reported that frailty increased the likelihood of emergency department visits [12]; the use of frailty-specific interventions may prevent ED readmissions [37]. ED visits have been less often examined in adverse outcomes predicted by frailty [6,7] and no consensus has been reached concerning ED visits and frailty status. These inconsistencies of the results concerning ED visits should be further investigated.

This study has some limitations. First, the primary study was carried out in a single center of an academic hospital, with a somewhat lower sample of hospitalized older adults admitted to the internal medical wards. During data collection in the primary study, number of inpatients and characterization of non-included hospitalized older adults were not collected, so is not possible to carry out the analysis between included and non- included participants. Additionally, the geographic location of this hospital might have contributed to the amount of frailty in the population. Therefore, these findings might have limited generalizability to other hospitals and units with hospitalized older adults. There is also the potential for a type II error due to multiple testing and modest sample size. Further, multicenter studies with larger samples are required to confirm our results. Second, functional decline was not assessed at hospital admission in the primary study. This could be relevant to better understand the functional trajectory of older adults and the predictive nature of frailty. Unlike our study, the majority of studies examined the difference between functional decline at admission and discharge, without considering baseline function. Thus, comparison with other studies is limited. Additionally, in the primary study, the Katz Index measured function and functional decline while others have employed tools such as the Barthel Scale; this could have an impact on the evaluation of the prevalence rate of functional decline. Third, the creation of the Frailty Index could limit the health deficits to be included and do not allow control of other potentially confounding variables. Despite this, the Frailty Index was created according to the best recommendations: 30 or more health deficits and the choice of these deficits met the criteria reported by Searle et al. [21]. We used cut-off value of 0.25 to determine presence of frailty. There is some controversy about this cut-off value, but it shows the strongest potential to predict adverse outcomes [5]. In our study, a pre-frailty condition was not considered, despite this category having been reported in other studies with FI [38,39]. However, in the systematic review, no difference was found on the risk of DF between frail and pre-frail older adults (RR = 0.88, 95% CI: 0.62:1.26) [6]. Finally, the AUC in the regression models range from 0.64 to 0.73; frailty associated with other variables explains approximately 70% of the DF. AUC values demonstrate acceptable discrimination.

## 5. Conclusions

This study showed a high prevalence of frailty among hospitalized older adults and demonstrated a higher risk for functional decline at discharge, 3 and 6 months, and more 30-day hospital readmission as well as more need for social support. We have demonstrated that frailty was an independent risk factor for functional decline at discharge and follow-up at 3 months. Strategies to screen and assess frailty as early as possible at admission coupled with targeted interventions to prevent or mitigate the progression of frailty may reduce functional decline during and after hospitalization, and should be explored further. Failure to address frailty as “geriatric vital sign” will potentially precipitate the cascade of functional decline.

## Figures and Tables

**Table 1:** Baseline characteristics of hospitalized older adults according to frailty status.

Sociodemographic Categorization	FIcat			FIcont	
	Non-Frail (*n* = 43) N (%)	Frail (*n* = 58) N (%)	Statistical Result	M ± SD	Statistical Result
**Gender**					
Male (*n* = 47)	24 (51.1%)	23 (48.9%)	X^2^(1) = 1.983 *p* = 0.159	0.23 ± 0.11	F(1;99) = 4.767 *p* = 0.031
Female (*n* = 54)	19 (35.2%)	35 (64.8%)		0.27 ± 0.10	
**Age**					
70–79 years (*n* = 35)	22 (62.9%)	13 (37.1%)	X^2^(2) = 9.797 *p* = 0.007	0.21 ± 0.11	F(2;98) = 5.327 *p* = 0.007
80–89 years (*n* = 52)	18 (34.6%)	34 (65.4%)		0.26 ± 0.10	
+90 (*n* = 14)	3 (21.4%)	11 (78.6%)		0.32 ± 0.10	
**Education Level**					
<basic education (*n* = 58)	19 (32.8%)	39 (67.2%)	X^2^(1) = 4.467 *p* = 0.035	0.27 ± 0.11	F(1;99) = 4.416 *p* = 0.038
≥basic education (*n* = 43)	24 (55.8%)	19 (44.2%)		0.22 ± 0.11	
**Marital status**					
Married (*n* = 46)	24 (52.2%)	22 (47.8%)	X^2^(1) = 2.888 *p* = 0.089	0.22 ± 0.11	F(1;98) = 6.535 *p* = 0.012
Not married (*n* = 54)	18 (33.3%)	36 (66.7%)		0.28 ± 0.11	
**Living status (baseline)**					
Spouse (*n* = 36)	20 (55.6%)	16 (44.4%)	X^2^(3) = 7.900 *p* = 0.048	0.21 ± 0.10	F(1;82) = 4.679 *p* = 0.004
Alone (*n* = 13)	7 (53.8%)	6 (46.2%)		0.22 ± 0.11	
Extended family (*n* = 35)	13 (37.1%)	22 (62.9%)		0.27 ± 0.10	
Nursing home (*n* = 17)	3 (17.6%)	14 (82.4%)		0.32 ± 0.11	
**Social support (Baseline)**					
With (*n* = 19)	7 (36.8%)	12 (63.2%)	X^2^(1) = 0.653 *p* = 0.419	0.27 ± 0.11	F(1;99) = 3.048 *p* = 0.085
Without (*n* = 65)	33 (50.8%)	32 (49.2%)		0.23 ± 0.10	
**Social support (at discharge)**					
With (*n* = 59)	27 (45.8%)	32 (54.2%)	X^2^(1) = 2.757 *p* = 0.097	0.25 ± 0.10	F(1;67) = 8.941 *p* = 0.004
Without (*n* = 10)	8 (80.0%)	2 (20.0%)		0.15 ± 0.08	
**Multimorbidity**					
With (*n* = 52)	14 (26.9%)	38 (73.1%)	X^2^(1) = 9.460 *p* = 0.002	0.28 ± 0.10	F(1;99) = 9.841 *p* = 0.002
Without (*n* = 49)	29 (59.2%)	20 (40.8%)		0.22 ± 0.11	
**Previous Hospitalization**					
Yes (*n* = 35)	9 (25.7)	26 (74.3)	X^2^(1) = 6.564 *p* = 0.012	0.29 ± 0.95	F(1;98) = 8.564 *p* = 0.004
No (65)	34 (52.3)	31 (47.7)		0.23 ± 0.11	
**Delirium at admission**					
With (*n* = 9)	2 (22.2%)	7 (77.8%)	X^2^(1) = 0.885 *p* = 0.347	0.31 ± 0.08	F(1;99) = 3.109 *p* = 0.081
Without (*n* = 92)	41 (44.6%)	51 (55.4%)		0.24 ± 0.11	
**ISAR-HP**					
Normal (*n* = 24)	22 (91.7%)	2 (8.3%)	X^2^(1) = 28.455 *p* < 0.001	0.14 ± 0.06	F(1;99) = 46.310 *p* < 0.001
At risk (*n* = 77)	21 (27.3%)	56 (72.7%)		0.28 ± 0.10	
	M ± SD	M ± SD	Statistical result	Correlation coefficient	
**Age**	79.49 ± 5.93	84.67 ±6.19	F(1;99) = 17.97 *p* < 0.001	0.390 *p*< 0.001	
**ISAR-HP**	1.95 ± 1.56	4.16 ± 1.18	F(1;99) = 65.29 *p* < 0.001	0.676 *p*< 0.001	
**Length of stay**	9.23 ±6.52	10.52 ±7.38	F(1;99) = 0.825 *p* = 0.366	0.136 *p* = 0.174	
**Number of medications**	5.70 ± 3.57	8.57 ± 3.79	F(1;99) = 14.910 *p* < 0.001	0.444 *p* < 0.001	

Note: FIcat—Frailty Index categorical; FIcont—Frailty Index continuous (higher scores indicate increasing frailty), ISAR-HP Identification of Seniors at Risk-Hospitalized Patients, M—Mean, SD—Standard Deviation.

**Table 2:** Outcomes based on frailty status at 3 months post-discharge.

3 Months Follow Up	FIcat			FIcont	
	Non-Frail (*n* = 41) N (%)	Frail (*n* = 52) N (%)	Statistical Result	M ± SD	Statistical Result
**Functional Decline**					
Discharge—Baseline					
Decline (*n* = 55)	17 (30.9%)	37 (69.1%)	X^2^(1) = 6.721 *p* = 0.010	0.29 ± 0.10	F(1;98) = 13.170 *p* < 0.001
No decline (*n* = 46)	26 (56.5%)	20 (43.5%)		0.21 ± 0.11	
3 m follow-up Discharge					
Decline (*n* = 19)	3 (15.8%)	16 (84.2%)	X^2^(1) = 6.381 *p* = 0.012	0.32 ± 0.08	F(1;91) = 8.322 *p* = 0.005
No decline (*n* = 74)	38 (51.4%)	36 (48.6%)		0.23 ± 0.11	
3 m follow-up—Baseline					
Decline (*n* = 33)	9 (26.5%)	24 (73.5%)	X^2^(1) = 5.322 *p* = 0.021	0.29 ± 0.09	F(1;92) = 10.060 *p* = 0.002
No decline (*n* = 60)	32 (53.3%)	28 (46.7%)		0.22 ± 0.11	
**Hospital readmission (90 days)**					
Yes (*n* = 20)	3 (15.0%)	17 (85.0%)	X^2^(1) = 6.651 *p* = 0.010	0.31 ± 0.09	F(1;94) = 7.402 *p* = 0.008
No (*n* = 76)	38 (50.0%)	38 (50.0%)		0.24 ± 0.11	
**ED visit**					
Yes (*n* = 38)	14 (36.8%)	19 (63.2%)	X^2^(1) = 0.532 *p* = 0.466	0.27 ± 0.11	F(1;57) = 1.371 *p* = 0.245
No (*n* = 58)	24 (46.6%)	31 (53.4%)		0.24 ± 0.11	
**Social Support**					
Yes (*n* = 47)	22 (46.8%)	25 (53.2%)	X^2^(1) = 0.885 *p* = 0.347	0.24 ± 0.10	F(1;57) = 12.170 *p* < 0.001
No (*n* = 12)	11 (91.7%)	1 (8.3%)		0.13 ± 0.06	

Note: ED—Emergency Department, FIcat—Frailty Index categorical; FIcont—Frailty Index continuous (higher scores indicate increasing frailty), ISAR-HP Identification of Seniors at Risk-Hospitalized Patients, M—Mean, SD—Standard Deviation.

**Table 3:** Outcomes based on frailty status at 6 months post-discharge.

6 Months Follow Up	FIcat			FIcont	
	Non-Frail (*n* = 38) N (%)	Frail (*n* = 49) N (%)	Statistical Result	M ± SD	Statistical Result
**Functional Decline**					
6 m follow-up—Baseline					
Decline (*n* = 33)	9 (27.3%)	24 (72.7%)	X^2^(1) = 4.792 *p* = 0.029	0.28 ± 0.09	F(1;85) = 15.358 *p* = 0.023
No decline (*n* = 54)	29 (53.7%)	25 (46.3%)		0.23 ± 0.12	
6 m follow-up—Discharge					
Decline (*n* = 21)	4 (19.0%)	17 (81.0%)	X^2^(1) = 5.571 *p* = 0.019	0.30 ± 0.09	F(1;85) = 6.195 *p* = 0.015
No decline (*n* = 66)	34 (51.5%)	32 (48.5%)		0.23 ± 0.11	
6 m and 3 m follow up					
Decline (*n* = 20)	4 (20.0%)	16 (80.0%)	X^2^(1) = 4.735 *p* = 0.030	0.30 ± 0.09	F(1;85) = 6.093 *p* = 0.016
No decline (*n* = 67)	34 (50.7%)	33 (49.3%)		0.23 ± 0.11	
**Hospital readmission (180 days)**					
Yes (*n* = 21)	8 (38.1%)	13 (61.9%)	X^2^(1) = 0.734 *p* = 0.115	0.26 ± 0.13	F(1;85) = 0.199 *p* = 0.657
No (*n* = 66)	30 (45.5%)	36 (54.5%)		0.25 ± 0.11	
**ED visit**					
Yes (*n* = 33)	14 (42.4%)	19 (57.6%)	X^2^(1) ≈ 0 *p* ≈ 1	0.25 ± 0.12	F(1;85) = 0.103 *p* = 0.749
No (*n* = 54)	24 (44.4%)	30 (55.6%)		0.25 ± 0.11	
**Social Support**					
Yes (*n* = 42)	21 (50.0%)	21 (50.0%)	X^2^(1) = 4.997 *p* = 0.025	0.24 ± 0.11	F(1;48) = 9.787 *p* = 0.003
No (*n* = 8)	8 (100.0%)	0 (0.0%)		0.12 ± 0.04	

Note: ED—Emergency Department, FIcat—Frailty Index categorical; FIcont—Frailty Index continuous (higher scores indicate increasing frailty), ISAR-HP Identification of Seniors at Risk-Hospitalized Patients, M—Mean, SD—Standard Deviation.

**Table 4:** Predictors for the FD between baseline–discharge.

FD between Discharge and Baseline	Univariable Models				Multivariable Model (*n* = 82)	
	No Decline	Decline	OR	[95% CI]	OR	[95% CI]
FIcat *						
Non-frail (ref)	26 (60.5)	17 (39.5)	1	-	-	-
Frail	20 (34.5)	38 (65.5)	2.91	[1.29–6.69]	-	-
**FIcont [0–100]**	21.0 ± 11.1	28.4 ± 9.7	1.07	[1.03–1.12]	1.07	[1.02–1.14]
**Gender**						
Masculine (ref)	27 (57.4)	20 (42.6)	1	-	1	-
Feminine	19 (35.2)	35 (64.8)	2.49	[1.12–5.63]	1.32	[0.51–3.47]
**Level of education**						
<Basic Education (ref)	24 (41.4)	34 (58.6)	1	-	-	-
≥Basic Education	22 (51.2)	21 (48.8)	0.67	[0.30–1.49]	-	-
**Marital status**						
Married (ref)	29 (63.0)	17 (37.0)	1	-	1	-
Not married	17 (31.5)	37 (68.5)	3.71	[1.64–8.69]	5.12	[1.79–16.23]
**Living condition (baseline)**						
Alone (ref)	4 (30.8)	9 (69.2)	1	-	-	-
Spouse	24 (66.7)	12 (33.3)	0.22	[0.05–0.83]	-	-
Extended family	11 (31.4)	24 (68.6)	0.97	[0.22–3.72]	-	-
Nursing home	7 (41.2)	10 (58.8)	0.63	[0.13–2.86]	-	-
**Social support (baseline)**						
Without (ref)	35 (53.8)	30 (46.2)	1	-	1	-
With	5 (26.3)	14 (73.7)	3.27	[1.11–11.01]	2.79	[0.92–9.31]
**Multimorbidity**						
Without (ref)	22 (44.9)	27 (55.1)	1	-	-	-
With	24 (46.2)	28 (53.8)	0.95	[0.43–2.08]	-	-
**Delirium (baseline)**						
Without (ref)	46 (50.0)	46 (50.0)	1	-	-	-
With	0 (0.0)	9 (100)	n.a.	-	-	-
**Previous hospitalization**						
Without(ref)	37 (56.9)	28 (43.1)	1	-	1	-
With	9 (25.7)	26 (74.3)	3.82	[1.59–9.82]	7.90	[2.71–26.78]
**Age (years)**	81.0 ± 6.1	83.6 ± 6.8	1.07	[1.00–1.14]	1.01	[0.94–1.10]
**Length of stay (days)**	9.0 ± 5.5	10.8 ± 8.0	1.04	[0.98–1.11]	-	-
**Number of medications**	6.8 ± 3.9	7.7 ± 4.0	1.07	[0.97–1.19]	-	-
					AUC = 0.73	

Note: CI—Confidence Interval; FIcat—Frailty Index categorical; FIcont—Frailty Index continuous (higher scores indicate increasing frailty), ISAR-HP—Identification of Seniors at Risk-Hospitalized Patients, M—Mean, OR—Odds Ratio, SD—Standard Deviation. * variable was not included in the multivariable model because of multicollinearity with FIcont (continuous) variable. n.a.: Not applicable due to an existence of value 0 in one or more cells.

**Table 5:** Predictors for the FD between 3-month follow-up and baseline.

FD between 3 Months Follow-Up and Baseline	Univariable Models				Multivariable Models (*n* = 93)	
	No Decline	Decline	OR	95% CI	OR	95% CI
FIcat *						
Non-frail (ref)	32 (78.0)	9 (22.0)	1	-	-	-
Frail	28 (53.8)	24 (46.2)	3.04	[1.25–7.95]	-	-
**FIcont [0–100]**	22.2 ± 11.4	29.5 ± 8.7	1.07	[1.02–1.12]	1.05	[1.01–1.09]
**Gender**						
Masculine (ref)	27 (61.4)	17 (38.6)	1	-	-	-
Feminine	33 (67.3)	16 (32.7)	0.77	[0.33–1.81]	-	-
**Level of education**						
<Basic Education (ref)	32 (59.3)	22 (40.7)	1	-	-	-
≥Basic Education	28 (71.8)	11 (28.2)	0.57	[0.23–1.36]	-	-
**Marital status**						
Married (ref)	28 (66.7)	14 (33.3)	1	-	-	-
Not married	31 (62.0)	19 (38.0)	1.23	[0.52–2.93]	-	-
**Living status (admission)**						
Alone (ref)	8 (66.7)	4 (33.3)	1	-	-	-
Spouse	24 (72.7)	9 (27.3)	0.75	[0.18–3.36]	-	-
Extended family	18 (56.3)	14 (43.8)	1.56	[0.40–6.82]	-	-
Nursing home	10 (62.5)	6 (37.5)	1.20	[0.25–6.12]	-	-
**Social support (baseline)**						
Without (ref)	42 (70.0)	18 (30.0)	1	-	-	-
With	9 (53.9)	8 (47.1)	2.07	[0.68–6.30]	-	-
**Multimorbidity**						
Without (ref)	31 (70.5)	13 (29.5)	1	-	-	-
With	29 (59.2)	20 (40.8)	1.64	[0.70–3.96]	-	-
**Delirium (baseline)**						
Without (ref)	54 (64.3)	30 (35.7)	1	-	-	-
With	6 (66.7)	3 (33.3)	0.90	[0.18–3.67]	-	-
**Previous hospitalization**						
Without (ref)	43 (70.5)	18 (29.5)	1	-	-	-
With	17 (54.8)	14 (45.2)	1.97	[0.80–4.86]	-	-
**Age (years)**	80.4 ± 6.3	85.4 ± 6.0	1.14	[1.06–1.24]	1.11	[1.04–1.20]
**Length of stay (days)**	8.5 ± 5.7	10.2 ± 6.3	1.05	[0.98–1.13]	-	-
**Number of medications**	7.0 ± 4.0	7.9 ± 3.8	1.06	[0.95–1.19]	-	-
					AUC = 0.68	

Note: CI—Confidence Interval; FIcat—Frailty Index categorical; FIcont—Frailty Index continuous (higher scores indicate increasing frailty), ISAR-HP—Identification of Seniors at Risk-Hospitalized Patients, M—Mean, OR—Odds Ratio, SD—Standard Deviation. * variable was not included in the multivariable model because of multicollinearity with FIcont (continuous) variable. n.a.: Not applicable due to an existence of value 0 in one or more cells.

**Table 6:** Predictors for the FD between 6-month follow-up and baseline.

FD between 6 Months Follow-Up and Baseline	Univariable Models				Multivariable Model (*n* = 71)	
	No Decline	Decline	OR	95% CI	OR	95% CI
FIcat *						
Non-frail (ref)	29 (76.3)	9 (23.7)	1	-	-	-
Frail	25 (51.0)	24 (49.0)	3.09	[1.25–8.19]	-	-
**FIcont [0–100]**	22.8 ± 11.9	28.3 ± 8.8	1.05	[1.01–1.10]	1.03	[0.99–1.09]
**Gender**						
Masculine (ref)	24 (58.5)	17 (41.5)	1	-	-	-
Feminine	30 (65.2)	16 (34.8)	0.75	[0.31–1.80]	-	-
**Level of education**						
<Basic Education (ref)	30 (60.0)	20 (40.0)	1	-	-	-
≥Basic Education	24 (64.9)	13 (35.1)	0.81	[0.33–1.95]	-	-
**Marital status**						
Married (ref)	24 (61.5)	18 (38.3)	1	-	-	-
Not married	29 (61.7)	18 (38.3)	0.99	[0.41–1.95]	-	-
**Living status (admission)**						
Alone (ref)	9 (75.0)	3 (25.0)	1	-	-	-
Spouse	22 (71.0)	9 (29.0)	1.23	[0.28–6.48]	-	-
Nursing home	13 (46.4)	15 (53.6)	3.46	[0.83–18.19]	-	-
Extended family	10 (62.5)	6 (37.5)	1.80	[0.36–10.60]	-	-
**Social support (baseline)**						
Without (ref)	39 (70.9)	16 (29.1)	1	-	1	-
With	6 (37.5)	10 (62.5)	4.06	[1.29–13.78]	3.61	[1.07–13.09]
**Multimorbidity**						
Without (ref)	27 (65.9)	14 (66.7)	1	-	-	-
With	27 (58.7)	19 (41.3)	1.36	[0.57–3.29]	-	-
**Delirium (baseline)**						
Without (ref)	51 (65.4)	27 (34.6)	1	-	-	-
With	3 (33.3)	6 (66.7)	3.78	[0.92–19.03]	-	-
**Previous hospitalization**						
Without (ref)	41 (68.3)	19 (31.7)	1	-	-	-
With	13 (48.1)	14 (51.9)	2.32	[0.92–5.98]	-	-
**Age (years)**	80.6 ± 6.3	83.9 ± 6.2	1.09	[1.01–1.17]	1.07	[0.99–1.18]
**Length of stay (days)**	8.6 ± 5.6	10.2 ± 6.2	1.05	[0.97–1.13]	-	-
**Number of medications**	6.8 ± 3.7	8.3 ± 4.4	1.10	[0.99–1.23]	-	-
					AUC = 0.64	

CI—Confidence Interval; FIcat—Frailty Index categorical; FIcont—Frailty Index continuous (higher scores indicate increasing frailty), ISAR-HP—Identification of Seniors at Risk-Hospitalized Patients, M—Mean, OR—Odds Ratio, SD—Standard Deviation. * variable was not included in the multivariable model because of multicollinearity with FIcont (continuous) variable. n.a.: Not applicable due to an existence of value 0 in one or more cells.

## Data Availability

The data presented in this study are available on request from the corresponding author. The data are not publicly available due to ethical restriction.

## Supplementary Materials

The following are available online at https://www.mdpi.com/article/10.3390/ijerph18137126/s1, Table S1: Variables/health-related deficits used to create the Frailty Index.
