# Supplementary material for: The Role of Frailty in Predicting 3 and 6 Months Functional Decline in Hospitalized Older Adults: Findings from a Secondary Analysis
Source: Int J Environ Res Public Health. 2021 Jul 3;18(13):7126. doi: 10.3390/ijerph18137126 (PMC8297187; doi:10.3390/ijerph18137126)
Supplement: Supplementary file 1 [file ijerph-18-07126-s001.zip › ijerph-1256782-supplementary.pdf]

Appendix table 1 - Variables/health-related deficits used to create the Frailty Index

Variables	**Operationalization**	**Score**
**1. Vision Loss**	Clinical characterization	Yes=1<br>No=0
**2. Hearing Loss**	Clinical characterization	Yes=1<br>No=0
**3. Myocardial infarction**	Charlson Comorbidity Index	Yes=1<br>No=0
**4. Congest Heart Failure**	Charlson Comorbidity Index	Yes=1<br>No=0
**5. Peripheral vascular disease**	Charlson Comorbidity Index	Yes=1<br>No=0
**6. Cerebrovascular accident**	Charlson Comorbidity Index	Yes=1<br>No=0
**7. Dementia**	Charlson Comorbidity Index	Yes=1<br>No=0
**8. Chronic Obstructive Pulmonary Disease**	Charlson Comorbidity Index	Yes=1<br>No=0
**9. Connective tissue disease**	Charlson Comorbidity Index	Yes=1<br>No=0
**10. Peptic ulcer disease**	Charlson Comorbidity Index	Yes=1<br>No=0
**11. Liver disease**	Charlson Comorbidity Index	Yes=1<br>No=0

**12. Moderate / severe chronic liver disease**	Charlson Comorbidity Index	Yes=1<br>No=0
**13. Diabetes mellitus**	Charlson Comorbidity Index	Yes=1<br>No=0
**14. Diabetes with end-organ damage**	Charlson Comorbidity Index	Yes=1<br>No=0
**15. Hemiplegia**	Charlson Comorbidity Index	Yes=1<br>No=0
**16. Moderate to severe Chronic Kidney Disease**	Charlson Comorbidity Index	Yes=1<br>No=0
**17. Malignant tumor**	Charlson Comorbidity Index	Yes=1<br>No=0
**18. Metastatic solid tumor**	Charlson Comorbidity Index	Yes=1<br>No=0
**19. Leukemia**	Charlson Comorbidity Index	Yes=1<br>No=0
**20. Musculoskeletal disorders**	Clinical History	Yes=1<br>No=0
**21. Diseases in the reproductive system**	Clinical History	Yes=1<br>No=0
**22. Diseases in the integumentary system**	Clinical History	Yes=1<br>No=0
**23. Help bathing**	Katz Index [1]	Dependence=1<br>Independence =0
**24. Help dressing**	Katz Index	Dependence=1

		Independence =0
**25. Help eating**	Katz Index	Dependence=1<br>Independence =0
**26. Help using toilet**	Katz Index	Dependence=1<br>Independence =0
**27. Help transferring**	Katz Index	Dependence=1<br>Independence =0
**28. Incontinent**	Katz Index	Dependence=1<br>Independence =0
**29. Need assistance for IADL**	ISAR-HP[2]	Yes=1<br>No=0
**30. Feel depressed**	Single item	Yes=1<br>No=0
**31. Sleep problems**	Single item	Yes=1<br>No=0
**32. Loss de balance**	- Physical restriction of mobility [3]	Sim=1<br>Não=0
**33. Cognitive decline**	Six-item cognitive impairment test (6CIT) [4]	Impairment (≥8)= 1<br>Normal (0-7)=0
**34. Weight loss**	Mini Nutricional Assessment [5]	Does not Know; Weight loss between 1and 3 kg or greater than 3 kg >3kg =1<br>No weight loss=0
**35. Fear of Falling**	Single item	Yes=1<br>No=0

**36. Fall risk**	Morse Fall Scale [6]	Low Risk (25-50) or High risk (≥51) = 1<br>No Risk (0-24) = 0
**37. Pressure injuries risk**	Braden scale [7]	Higher risk (≤16) = 1<br>Lowe risk (≥ 17) =0
**38. Orientation**	Confusion Assessment Method [8]	Yes=1<br>No=0
**39. Polypharmacy**	Patient medication records	>4 medications =1<br>≤ 4 medications =0
**40. Psychotropic drugs**	Patient medication records	Yes=1<br>No=0

IADL Instrumental activities of daily living

References

1. Duque, S.; Gruner, H.; Clara, J.; Ermida, J.; Veríssimo, M. Avaliação Geriátrica. Portugal: Núcleo de Estudos de Geriatria da Sociedade Portuguesa de Medicina Interna (GERMI) Available online: http://www.spmi.pt/docs_nucleos/GERMI_36.pdf.
2. Tavares, J.; Grácio, J.; Nunes, L. Predictive validity of the Identification of Seniors at Risk - Hospitalized Patient tool foridentifying functional decline. *Rev. Enferm. Ref.* **2017**, *IV Série*, 145–154, doi:10.12707/RIV17049.
3. Faria, H.; Paiva, A.; Marques, P. A restrição física da mobilidade – estudo sobre os aspetos ligados à sua utilização com fins terapêuticos. *Rev. Enferm. Ref.* **2012**, *III Série*, 7–16, doi:10.12707/RIII1192.
4. Apóstolo, J.L.A.; Paiva, D.D.S.; Silva, R.C.G. da; Santos, E.J.F. Dos; Schultz, T.J. Adaptation and validation into Portuguese language of the six-item cognitive impairment test (6CIT). *Aging Ment. Health* **2018**, *22*, 1190–1195, doi:10.1080/13607863.2017.1348473.
5. Loureiro, H. Validação do mini-nutritional assesment em idosos. Master´s Thesis, Repositório científico da UC, 2008.
6. Costa-Dias, M.; Ferreira, P.; Oliveira, A. Adaptação cultural e linguística e validação da Escala de Quedas de Morse. *Rev. Enferm. Ref.* **2014**, *IV Série*, 7–17, doi:10.12707/RIII1382.
7. Direção-Geral da Saúde Escala de Braden: Versão Adulto e Pediátrica (Braden Q) Available online: https://nocs.pt/escala-braden/.

8. Sampaio, F.; Sequeira, C. Tradução e validação do Confusion Assessment Method para a população portuguesa. *Rev. Enferm. Ref.* **2013**, *III Série*, 125–134, doi:10.12707/RIII12127.
